# Severe chest allodynia as an unusual first presentation of hydatid disease: a case report

**DOI:** 10.1186/s12879-019-3670-7

**Published:** 2019-01-09

**Authors:** Flaminia Coluzzi, Roberto Luca Meniconi, Damiano Caruso, Flaminia Rivosecchi, Linda Petrone, Delia Goletti, Giuseppe Maria Ettorre

**Affiliations:** 1 Department of Medical and Surgical Sciences and Biotechnologies, Sapienza University of Rome, Latina, Italy; 2 Division of General Surgery and Liver Transplantation, POIT Department, San Camillo Hospital – “Lazzaro Spallanzani” National Institute for Infectious Diseases (INMI)-IRCCS, Rome, Italy; 3 Department of Radiological Sciences, Oncology and Pathology, Sapienza University of Rome, Rome, Italy; 4 Department of Epidemiology and Preclinical Research, Translational Research Unit, “Lazzaro Spallanzani” National Institute for Infectious Diseases (INMI)- IRCCS, Rome, Italy

**Keywords:** Allodynia, Chest pain, *Echinococcus granulosus*, Hydatid cyst, Liver cyst, Retroperitoneum

## Abstract

**Background:**

Cystic echinococcosis (CE) is a worldwide zoonosis and the liver is the most commonly affected organ. Clinical manifestations range from completely asymptomatic cysts to a potential lethal cyst rupture and anaphylaxis.

**Case presentation:**

Severe chest allodynia was an unusual clinical presentation of hepatic cyst rupture in the retroperitoneal space, without any other specific symptoms. CE diagnosis was confirmed by computed tomography scan and magnetic resonance. The patient underwent hepatectomy with complete resolution of the neuropathic pain.

**Conclusions:**

Retroperitoneal hydatid cyst rupture is a rare event and its clinical manifestation may mimic other chest neuropathies.

## Background

Cystic echinococcosis (CE) is a zoonosis caused by the tapeworm *Echinococcus granulosus* sensu *lato.* More than 1 million people have been estimated to be infected worldwide [1] and Italy, as well as the countries of the Mediterranean basin, are considered endemic [2, 3]. In Italy, the estimated incidence of hospitalized cases in 2014 is up to 1.06/100,000 inhabitants/year [4], but likely these data are underestimated.

The most common location of the echinococcal cysts is the liver, but cysts can be found in almost any organ including lungs, spleen, and kidney. Their clinical manifestations range from asymptomatic to severe, potentially lethal manifestations, such as in case of cyst rupture and anaphylaxis. Imaging, mainly ultrasound (US), is the basis of CE diagnosis and clinical management whereas serology is complementary. Current serological tests present several limits, including low sensitivity. Moreover, serology does not help clinical decision during follow-up, as it does not allow assessing cyst biological viability. Recently, an assay based on the detection of Interleukin (IL)-4 in response to the Antigen (AgB; native protein or peptides) has been shown to be useful for active-viable cysts identification [5, 6].

We present a case of a hepatic hydatid cyst complicated by rupture in the retroperitoneal space, with the atypical symptom of chest allodynia and right hypochondrial discomfort.

Retroperitoneal involvement by hydatid disease is very rare with only a few published cases [7–9]. We report for the first time a case of retroperitoneal rupture of a hepatic hydatid cyst, with neuropathic thoracic pain being the main presenting symptom. Chest pain may be related to many differential diagnosis, with possible misleading diagnosis. However, as rupture of CE requires a prompt approach to avoid potentially severe or even life threatening complications, a quick and precise diagnosis is imperative together with referral to a specialized center, without delays in the diagnosis and treatment.

Furthermore, this case illustrates that evaluation of calcification extent, although used for the staging of CE cysts, cannot be used as the only feature, as signs of calcification may coexist with still metabolically active cysts [10]. Immunological tests may help to define cyst activity.

## Case presentation

A 71-year-old woman presented to the Division of General Surgery and Liver Transplantation, POIT Department, San Camillo Hospital – “Lazzaro Spallanzani” National Institute for Infectious Diseases (INMI) in Rome with the acute onset of severe burning sensation for 1 week, referred to the right chest wall and the back, graded as a 8 on a 0 to 10 numeric rating scale. The pain was worsening with deep breathing and during walking. The patient complained of occasional, sharp, moderate, right upper quadrant abdominal discomfort and flu-like symptoms, such as fatigue, low-grade fever, night sweats and generalize bony pains. The treatment with acetaminophen 1 g three times daily and pregabalin 75 mg twice daily for 5 days, prescribed by her general practitioner, was not successful. Medical history included current mild hypertension and hypothyroidism, under pharmacological treatment, chickenpox at the age of 40 years, and an episode of acute pericarditis and pleuritis 10 years before. The patient was retired, living in Rome and owner of three dogs. However, during childhood, she spent 10 years in a rural area in central Italy (the region of Abruzzo), where contact with animals was common. Of note, the patient’s father underwent thoracic surgery for pulmonary hydatid cyst.

On physical exam, blood pressure was 120/75 mmHg, heart rate 90 beats per minute, and respiratory rate 16 breaths per minute. Heart, lung and abdomen findings were within normal limits with the exception of tenderness upon palpation of the right upper abdominal quadrant. Murphy’s and Blumberg’s signs were negative. Allodynia and hyperesthesia upon palpation of the right chest wall were observed, without any identifiable neural root involvement.

Routine laboratory work up showed White Blood Cell (WBC) count 9960/mm3, with eosinophil count 360 /mm3 (3.7%) and neutrophil count 7120 /mm3 (71.5%), platelets count (plt) 352,000/mm3, hemoglobin 13.3 g/dl, erythrocyte sedimentation rate (ESR) 33 mm/hr., and C-reactive protein (CRP) 98.7 mg/L. Total protein count was in the normal range (7.0 g/dL), with some alterations of the electrophoretic profile, including increased alpha-1 and alpha-2 protein [0.56 (8%) and 1.12 (16%) g/dL3, respectively]. Hepatic enzymes profile was unremarkable with a γ-glutamyl tranferase (GGT) of 33 Us/L, and both Alanine Aminotransferase (ALT) and Aspartate Aminotransferase (AST) of 19 Us/L. No alterations of amylase and lipase levels were detected.

**Fig. 1:**
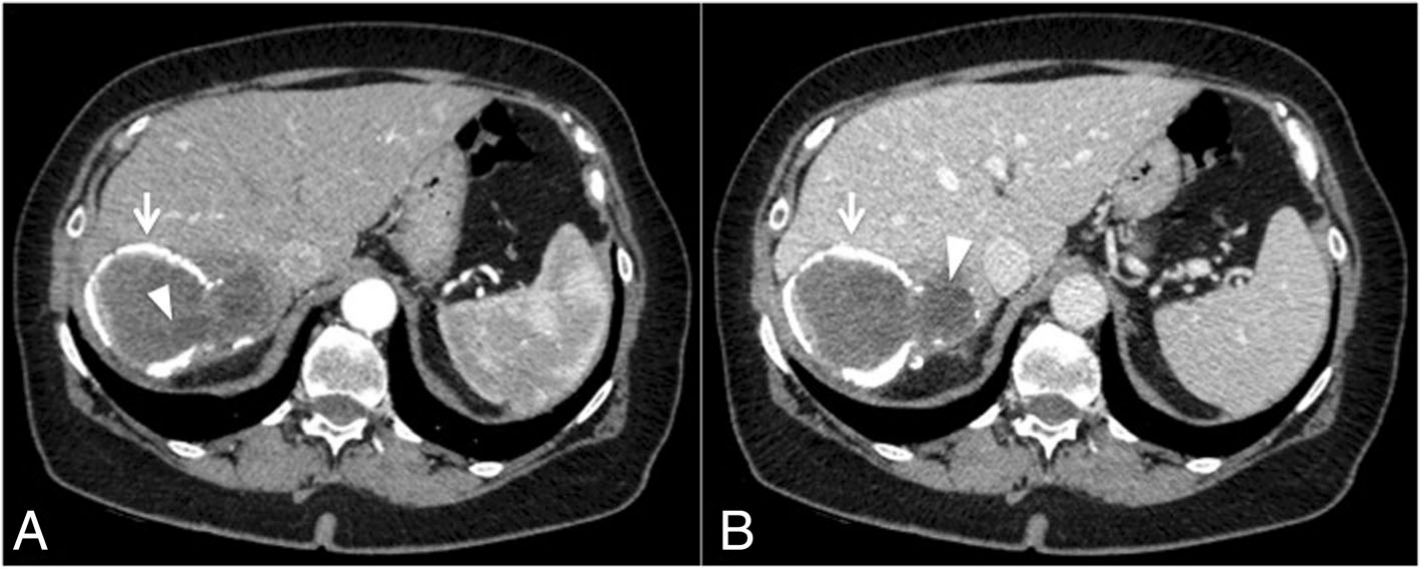
Axial CT view after intravenous contrast media administration during arterial (**a**) and venous (**b**) phase, shows a big cystic lesion of the liver, at segment VII, with subtotally calcified walls (arrows) and internal daughter cysts (arrow-head)

**Fig. 2:**
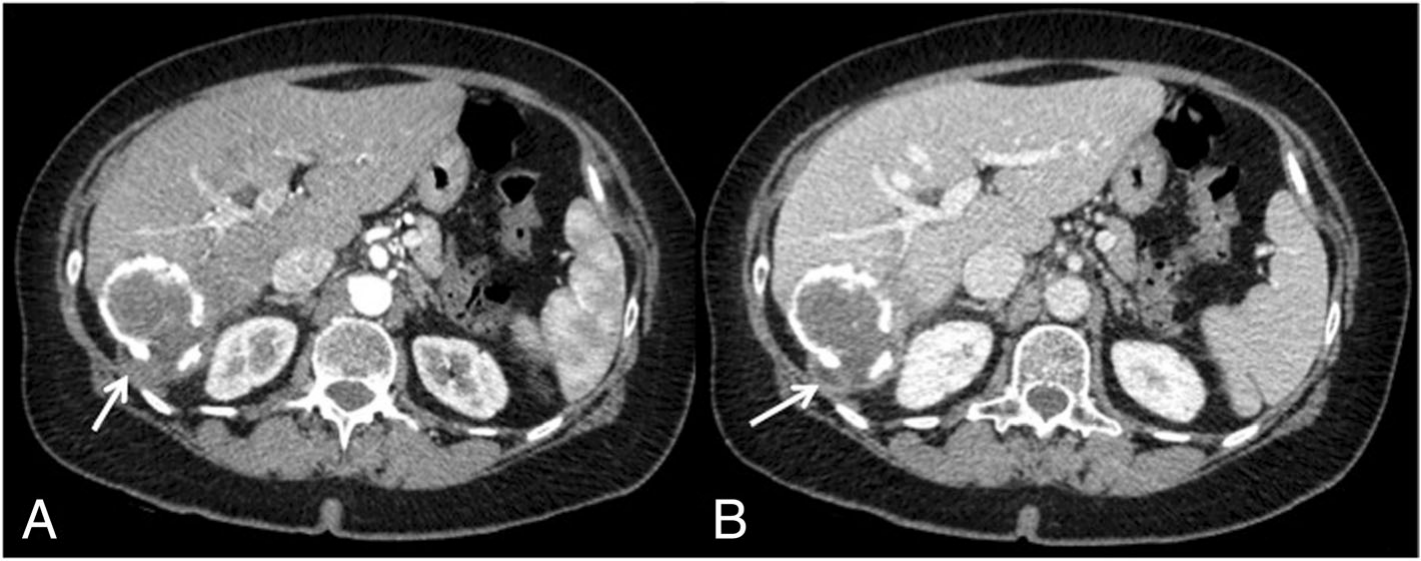
Axial CT view during arterial (**a**) and venous (**b**) phase. An incomplete wall is seen along the posterior side of the cyst in connection with an hypoattenuating area corresponding to extrahepatic extravasation of the cyst contents (arrows) due to extrahepatic rupture of the hydatid cyst

**Fig. 3:**
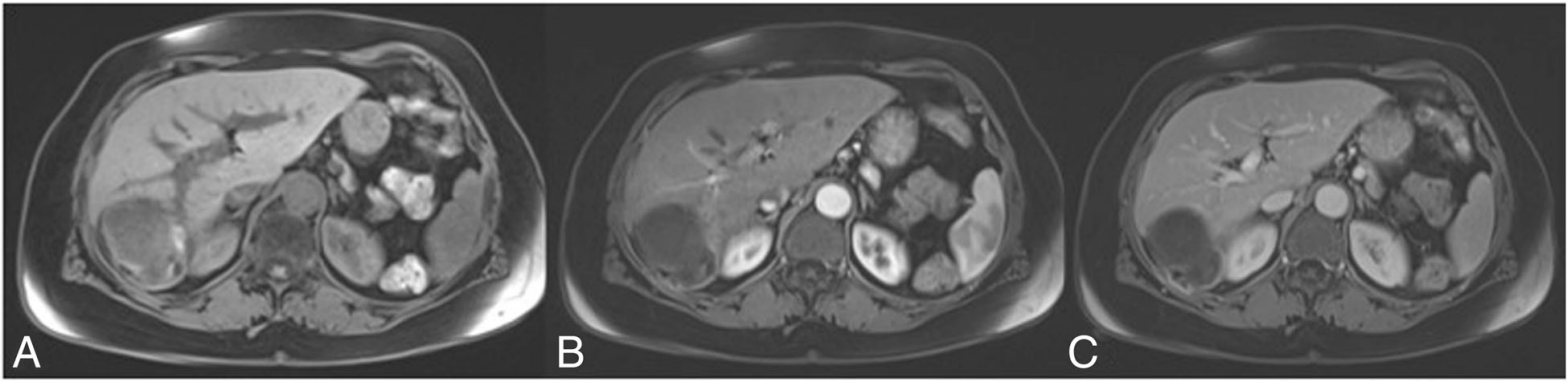
FS-T1-weighted pre (**a**) and post intravenous contrast injection (**b**, arterial phase and **c**, venous phase) MR images confirmed the hydatid hepatic cyst at the VII segment

Abdominal CT scan showed a thick walled cystic lesion with egg-shell calcification in the right lobe of the liver measuring about 93 × 70 mm (Fig. 1). The wall of the cyst showed discontinuity from which fluid leaked outside the cyst wall and the liver capsule into the retroperitoneum, with initial compression of the right kidney (Fig. 2). A contrast-enhanced MRI of the upper abdomen confirmed the presence of a cystic lesion in the right hepatic lobe (Fig. 3).

**Fig. 4:**
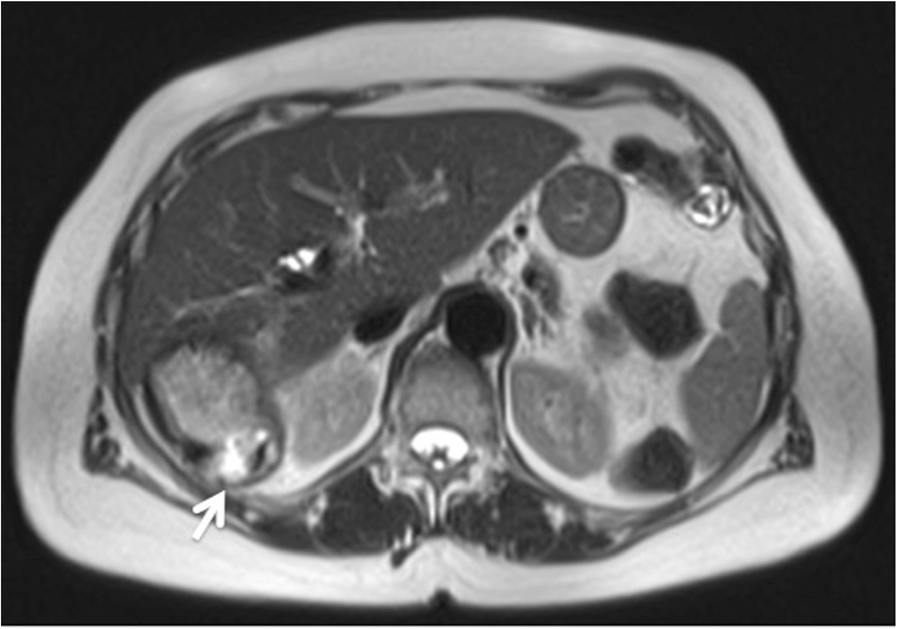
T2 weighted image shows the interruption of the hypointense outer ring, representing the pericyst and the extravasation of the cyst contents (arrow)

An interruption in the posterior face of the wall, hypointense in T2 weighted image, was visualized, confirming the cyst rupture and the extravasation of the cyst contents (Fig. 4).

Serology for *Echinococcus granulosus* (commercial enzyme-linked immunosorbent assay confirmed by western blot) was positive with both tests. In addition, an experimental test based on IL-4 detection after whole blood stimulation with AgB peptides was performed. The IL-4 level was 0.62 pg/mL, suggesting the presence of viable cysts based on the previously identified cut-off of > 0.59 pg/mL in serology-positive patients [6].

**Fig. 5:**
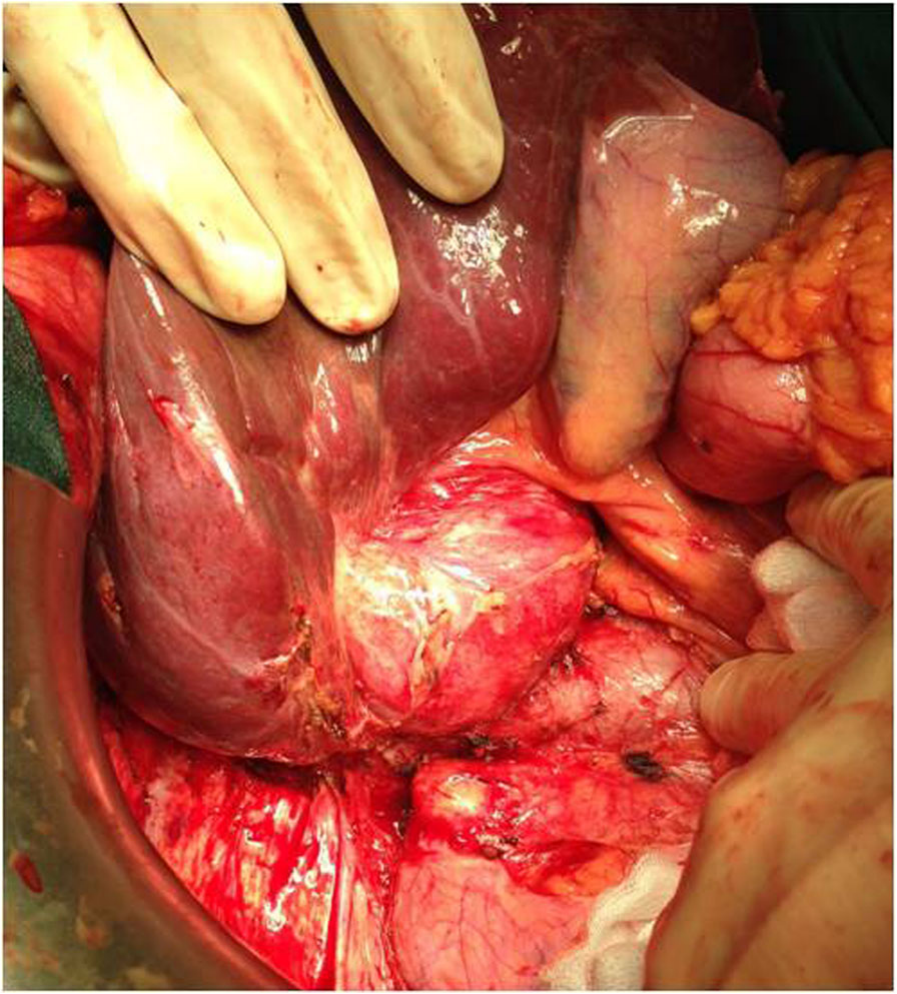
Intraoperative view of the echinococcal cyst in the right posterior sector of the liver with retroperitoneal involvement

**Fig. 6:**
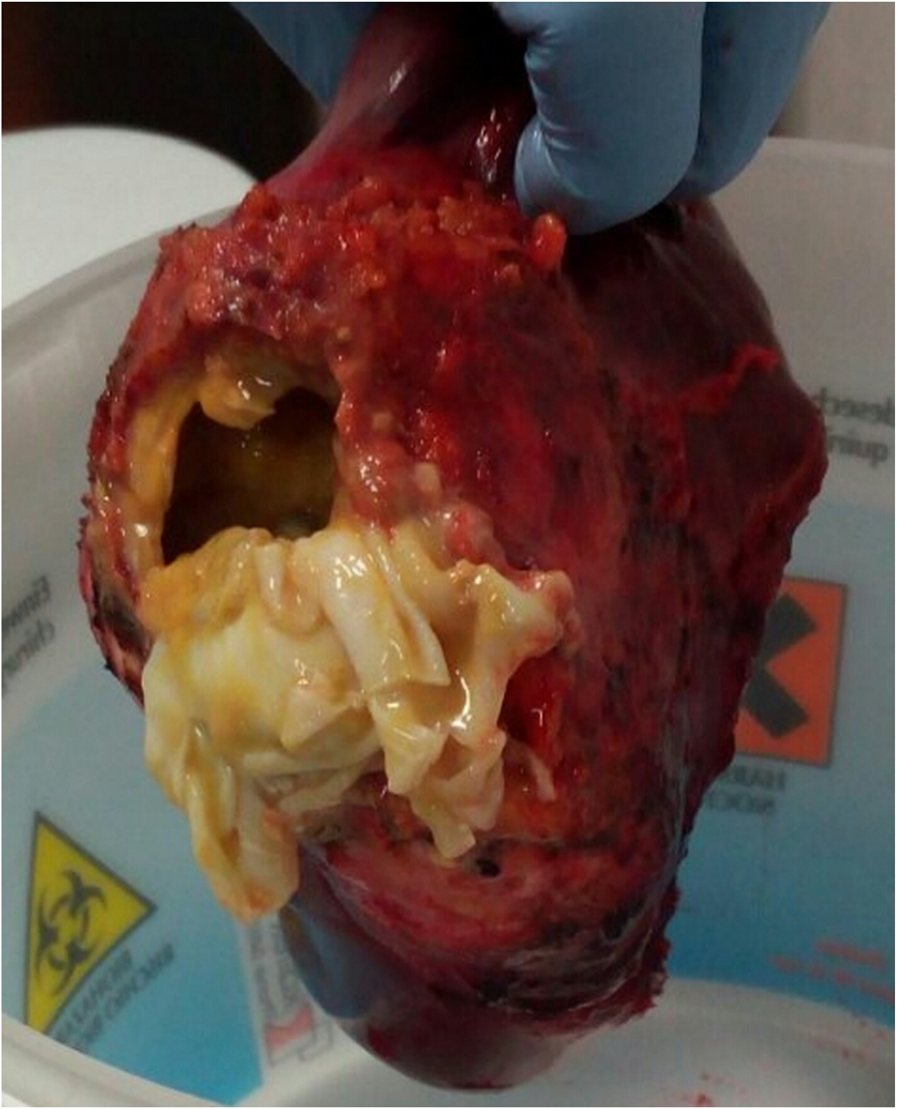
Hydatid cyst with many daughter vesicles

Based on the findings, the diagnosis was spontaneous rupture of hepatic hydatid cyst, as no history of major trauma was reported. No symptoms of anaphylaxis (rash, laryngeal edema, bronchospasm, severe hypotension) were detected. Treatment with oral albendazole (400 mg twice daily) was started immediately, and the patient underwent right hepatectomy and the operatory field was protected with 20% hypertonic saline to help preventing secondary echinococcosis. Macroscopically the cyst contained many daughter vesicles, compatible with a CE2 stage (Figs. 5 and 6). Surgery resulted in the complete resolution of chest allodynia.

One month after operation the physical conditions of the patient were good. The treatment with albendazole was continued for additional 6 months.

Written informed consent for publication of clinical details and clinical images were obtained from the patient. A copy of the consent form is available for review by the Editor of this journal.

## Discussion and conclusions

We describe a case of atypical presentation of spontaneous rupture of a hepatic CE cyst. Chest pain is a common complaint and reason for consultation in primary care. This may be due either to visceral disease, including pleural, gallbladder, cardiac and vascular conditions, or to diseases of the chest wall, such as musculoskeletal diseases, radicular pain, local neuritis (herpes zoster), vertebral body involvement by inflammation, infectious disease (tuberculosis) or tumors, and fractured ribs. As chickenpox was reported in the medical history, chest allodynia was associated to herpes zoster neuralgia, and gabapentinoids/acetaminophen treatment was prescribed before hospital admission. However, the absence of vesicles after 1 week of acute pain onset and the inflammation status found on laboratory work up, led to further investigations. Imaging results suggested the diagnosis of a retroperitoneal rupture of a hepatic hydatid cyst, which is a rare event and an unusual cause of chest allodynia. Moreover, serology and the experimental whole blood testing confirmed the diagnosis of cystic echinococcosis.

The CT images showed a calcification of the cyst wall. This is characteristic of CE, but is not a feature restricted to CE5 and CE4 stages; it may be present across all cystic stages and therefore cannot be considered indicative of the death of the cyst [11]. Cyst stage is easily assessable by appropriate imaging (US or MRI; less for other techniques) but the stadiation does not always matches with the cyst biological viability [12]. This applies particularly to CE3a cysts (50% apparently are non viable and 50% viable) and to CE4 cysts, as a proportion of cysts which appear inactive on imaging are actually biologically viable, especially those reaching the CE4 stage after treatment [12–14]. Indeed, despite the thick calcific wall, in our patient the cyst rupture was spontaneous, and the content structure did indicate a viable stage.

Recently, a whole-blood assay based on AgB synthetic peptides has been suggested to provide information on the cyst biological viability, mainly in CE patients with a positive serology [6]. The experimental whole blood test in our patient suggested the biological activity of the cyst and the microscope detection of protoscoleces from the cyst fluid confirmed its viable nature.

Surgery maintains a key role in the management of complicated cysts [15]. Surgery strategy depends on the cyst stage, localization and relationship between the cyst and vascular or biliary structures, ranging from liver resection to radical pericystectomy or de-roofing of the cyst. In our case, a right hepatectomy was performed because of the involvement of the right posterior bile duct that could increase the risks of postoperative bile leakage after a simple pericystectomy.

Primary retroperitoneal hydatid cysts have been described in literature, however this is the first reported case of hepatic echinococcosis with rupture of the cyst into the retroperitoneal space, presenting as severe chest allodynia. Moreover, spontaneous cyst rupture is rare and the lack of traumatic events may induce the clinician to exclude a priori a diagnosis of CE. However, CE is endemic in Italy and in the region of Abruzzo where the patient spent her childhood. Abruzzo has been recognized as the third Italian region for CE prevalence after Sicily and Calabria, as shown by Hospital Discharge Records and Italian Register of CE (Registro Italiano Echinococcosi Cistica, RIEC, now European ERCE) data [16, 17]. Therefore, in endemic areas, in presence of symptoms suggestive of CE, in a patient with a history of exposure to animal carriers and/or with relatives affected by this disease, hydatidosis should always be included in the differential diagnosis of cystic lesions of the liver. The presentation of a cystic rupture is often an emergency, because the content not only causes chemical peritonitis but also anaphylaxis and secondary echinococcosis. However, as described in our case report, the presentation can be atypical and the involvement of the retroperitoneal space may cause chest allodynia, mimicking other pathological conditions.

## Abbreviations

CE: Cystic echinococcosis
CT: Computed tomography scan
MRI: Magnetic resonance imaging
